# Heterogeneity in the psychosocial and behavioral responses associated with a diagnosis of suspected Lynch syndrome in women with endometrial cancer

**DOI:** 10.1186/s13053-022-00233-1

**Published:** 2022-07-15

**Authors:** Sowmya Jonnagadla, Sharelle L. Joseland, Sibel Saya, Nicole den Elzen, Joanne Isbister, Ingrid M. Winship, Daniel D. Buchanan

**Affiliations:** 1 Department of Clinical Pathology, Colorectal Oncogenomics Group, Melbourne Medical School, The University of Melbourne, Victorian Comprehensive Cancer Centre, 305 Grattan Street, Parkville, Victoria 3010 Australia; 2 The University of Melbourne Centre for Cancer Research, Victorian Comprehensive Cancer Centre, Parkville, Victoria 3000 Australia; 3 Department of Paediatrics, The University of Melbourne, Parkville, Victoria 3010 Australia; 4 Department of General Practice, The University of Melbourne, Parkville, Victoria 3010 Australia; 5 Genomic Medicine and Family Cancer Clinic, The Royal Melbourne Hospital, Parkville, Victoria 3050 Australia; 6 Department of Medicine, The Royal Melbourne Hospital, Parkville, Victoria 3050 Australia

**Keywords:** Suspected Lynch syndrome, Lynch-like syndrome, Psychosocial, Endometrial cancer, DNA mismatch repair, Cancer screening

## Abstract

**Background:**

A suspected Lynch syndrome (SLS) diagnosis is made when a tumor exhibits DNA mismatch repair deficiency but cannot be definitively assigned to an inherited or non-inherited etiology. This diagnosis poses challenges for healthcare professionals, patients, and their families in managing future cancer risks and clinical care.

**Methods:**

This qualitative study aimed to explore the psychosocial and behavioral responses of endometrial cancer (EC) patients receiving a SLS diagnosis (EC-SLS). Semi-structured telephone interviews were conducted with 15 EC-SLS women, transcribed, and thematically analyzed.

**Results:**

Most who interpreted their result as negative for Lynch syndrome (LS) believed they were at population-level risk of cancer and felt happy and relieved. Many participants who interpreted their result as inconclusive/not definitive for LS were confused about their cancer risk and experienced negative emotions of anger and frustration. Despite variation in colorectal cancer screening recommendations reported by participants, most adhered to the advice given. Almost all participants communicated their genetic test result to immediate family members; however, communication of family cancer risk management advice was more limited due to most participants reporting not receiving family screening advice. A family history of cancer and a professional healthcare background influenced participants’ engagement in regular cancer screening.

**Conclusion:**

These findings highlight variability in the psychosocial and behavioral responses associated with EC-SLS, providing insight into how healthcare professionals can optimally manage and support such individuals.

**Supplementary Information:**

The online version contains supplementary material available at 10.1186/s13053-022-00233-1.

## Background

Lynch syndrome (LS) is a highly penetrant, autosomal dominant cancer-predisposing condition [1] caused by germline pathogenic variants in the DNA mismatch repair (MMR) genes (*MLH1*, *MSH2*, *MSH6* and *PMS2*), or the *EPCAM* gene [2]. People with LS inherit an elevated risk of certain cancers, particularly colorectal cancer (CRC) and endometrial cancer (EC) [3], as well as ovarian, stomach, small intestinal, breast, pancreatic, prostate, urinary tract, brain, skin and hepatobiliary tract cancer [4, 5].

Genetic testing (GT) for LS is offered to individuals diagnosed with colorectal or endometrial tumors that display MMR-deficiency, visualized by MMR protein immunohistochemistry (IHC), and/or high microsatellite instability [6, 7]. Australian guidelines recommend that individuals with LS undertake annual to biennial colonoscopies from the age of 25 and consume low-dose aspirin from age 30 [8]. Biennial gastroscopies are also recommended for individuals with a family history or high ethic risk of gastric cancer [8]. Post-childbearing, women with LS are recommended to have a hysterectomy and to consider a salpingo-oophorectomy to reduce the risk of EC and ovarian cancer respectively [8].

A suspected Lynch syndrome (SLS) diagnosis is made when a tumor exhibits MMR-deficiency but cannot be definitively assigned to an inherited or non-inherited etiology with current testing approaches. Up to 70% of individuals with MMR-deficient tumors lacking *MLH1* hypermethylation receive an uninformative GT result – either negative or variant of uncertain significance (VUS) – resulting in an SLS diagnosis [9, 10]. Recent studies found that approximately 69% of patients with SLS presented with double somatic mutations of MMR genes [11–13].

There is evidence that individuals with SLS are at an intermediate lifetime risk of CRC and EC compared with individuals with LS and the general population [10, 14]. Moreover, the average age of onset of cancers in individuals with SLS is similar to that of LS cancers [9, 15], or between that of LS and sporadic cancers [10]. Research suggests that first-degree relatives of individuals with SLS are also at an intermediate risk of CRC and EC [10, 16]. However, there are no cancer risk management guidelines in Australia for individuals with SLS or their first-degree relatives [17, 18].

There is limited research on patients’ psychosocial and behavioral responses to an SLS diagnosis, the majority of which has been conducted on CRC patients. Studies that quantified cancer screening adherence among SLS individuals reported high colonoscopy screening adherence (48–85%) with screening frequency ranging from annual to triennial [19, 20]. These studies also showed a high rate (77%) of surgery to prevent the risk of EC and/or ovarian cancer [20]. Katz, Burton-Chase [19] reported that many SLS-CRC patients in their cohort perceived that they and their family were at high risk of CRC and LS. They also found that 59% of participants did not understand their result as uninformative negative or uncertain. The authors also reported that most of their cohort disclosed their GT results and CRC screening recommendations to at least one first-degree relative, but rarely to extended family members [19, 21].

A SLS diagnosis poses challenges for healthcare professionals (HCPs), genetic counselors (GCs), patients, and their families around the uncertainty of the diagnosis and managing future cancer risks. Importantly, it is not known whether the findings of studies on SLS-CRC patients particularly regarding colonoscopy screening, translate to EC-SLS patients. Previous studies have also all been based in North America and primarily used quantitative methodologies. Hence, this qualitative study is the first of its kind to explore the cancer screening behaviors, understanding of GT results, psychosocial responses, cancer risk perceptions and family communication among EC-SLS patients.

## Methods

### Participants and recruitment

This study was approved by The University of Melbourne Human Research Ethics Committee (Ethics approval number: 1955697). Eligible participants were recruited from the ANGELS (Applying novel genomic approaches to early-onset and suspected Lynch syndrome colorectal and endometrial cancers) study that enrolls participants from 16 cancer genetics services across Australia. Prior to joining the ANGELS study, all participants had experienced GT for LS and genetic counselling by a range of different GCs within these 16 cancer genetics services. Participants were purposively sampled to capture a wide range of experiences by incorporating diversity of age, ethnicity, geographic location within Australia, and histopathological tumor results. Participants were eligible for the study if they were: (a) 18 years and above; (b) diagnosed with EC and tumor testing showed loss of expression (LOE) of one or more MMR proteins by IHC (MMR-deficiency); (c) a negative or inconclusive result for tumor *MLH1* gene promoter hypermethylation for ECs exhibiting LOE of MLH1 and/or PMS2; (d) tested and received an uninformative germline GT result for MMR and *EPCAM* genes and no VUS finding; (e) able to speak English and provide informed consent; and (f) not in a palliative stage or significantly unwell.

### Data collection

Semi-structured interviews were conducted to investigate participants’ experiences of an SLS diagnosis and gather data rich in experience and emotion [22]. A semi-structured interview guide was chosen to collect open-ended data and to explore participants’ thoughts, feelings, and beliefs of receiving a diagnosis of SLS. Fifteen telephone interviews were conducted by a single interviewer (SJ) using an interview guide that was developed from a literature review and the clinical expertise of the research team. The interview guide included open-ended questions that covered five topics, including understanding of cancer diagnosis and GT results, cancer risk perception for themselves and their family, psychosocial response to GT results, cancer risk management and screening behaviors, and family communication of GT results and cancer risk management advice. The interview guide was supplemented by any themes arising from analysis of early interviews. All interviews were audio-recorded, transcribed verbatim and deidentified using pseudonyms. Participant demographic details were obtained from ANGELS study records and participant-reported information.

### Data analysis

Thematic analysis was undertaken on QSR International’s NVivo 12 software [23]. Inductive coding was used to categorize similarities and differences among participant responses [24]. Codes with shared meaning enabled the development of themes relating to study aims [25]. A subset of transcripts was independently coded by research team members (SLJ, SS, JI), which facilitated rigor of analysis [26, 27]. Thematic saturation was achieved after 12 interviews, following which no new themes emerged [28].

### Ethics approval

Approval to conduct this human subject’s research was obtained by The University of Melbourne Human Research Ethics Committee (Ethics approval number: 1955697) on December 10, 2019. All procedures followed were in accordance with the ethical standards of the responsible committee on human experimentation (institutional and national) and with the Helsinki Declaration of 1975, as revised in 2000.

## Results

### Participants

Participant demographics are described in Table 1. All 16 invited participants agreed and consented to participate. However, one participant declined to be interviewed due to personal reasons (Response rate = 93.75%). Fifteen participant interviews were conducted between March and July 2020 and ranged from 35 to 129 minutes, with a mean duration of 60 minutes. Participants came from five Australian states and territories and ranged in age from 43 to 75 years old. All but one participant had at least one first-degree relative diagnosed with cancer. Purposive sampling enabled recruitment of individuals with diversity in terms of age, ethnicity, geographic location within Australia, EC staging and tumor MMR IHC results.

**Table 1:** Participant demographics (*n* = 15)

Criteria	Range / Categories (numbers)	Mean / Percentage
Age ^a^	43–75 years	64 years
Age at EC diagnosis	41–72 years	60 years
Time since EC diagnosis	1–9 years	3.4 years
Time since genetic test result	0–2.4 years	1.3 years
States covered within Australia	Western Australia – 3	20%
	Queensland – 2	13.33%
	New South Wales – 2	13.33%
	Australian Capital Territory – 2	13.33%
	Victoria – 6	40%
Tumor MMR IHC results ^b^	LOE MLH1/PMS2–9	60%
	LOE MSH2/MSH6–4	26.67%
	LOE MSH6–2	13.33%
Highest level of education	School level – 7	46.67%
	Bachelor’s Degree – 5	33.33%
	Master’s Degree – 3	20%
Occupation ^c^	Nurse – 6	40%
	Other healthcare – 2	13.33%
	Other – 7	46.67%
Ethnicity	Caucasian – 13	86.67%
	Other – 2	13.33%
Stage of EC ^d^	Stage 1–7	46.67%
	Stage 2–5	33.33%
	Stage 3–3	20%
Personal history of other cancers ^e^	Breast cancer - 2	13.33%
	Bowel cancer - 1	6.67%
Family history of cancer ^f^	≥1 first degree relative – 14	93.33%
	≥ 1 second or third degree relative – 13	86.67%

^a^ Age – at the time of study interview

^b^ LOE – Loss of Expression of gene in somatic tumor testing

^c^ Other occupations include accounts, insurance, journalism, government and education

^d^ FIGO staging in endometrial carcinomas [29]

^e^ Personal history of other primary cancers before the EC diagnosis

^f^ Family history of cancers included cancer of the uterus, bowel, brain, ureter, stomach, breast, ovary, lungs, throat, skin, prostate, liver, blood and pancreas

*Abbreviations*: *EC* Endometrial cancer, *MMR* Mismatch-repair, *IHC* Immunohistochemistry, *LOE* Loss of expression

### Theme 1: Interpretation of GT results influences cancer risk perception and psychosocial impacts of testing

Participants interpreted their GT results as either negative for LS or inconclusive/not definitive for LS. This interpretation influenced the psychosocial impacts of testing and their perceptions of their and their family’s future cancer risk.

#### Interpretation of genetic test results

Over half of the participants understood that they received negative results from GT, and most of these participants reported that they were negative for LS.

*Just that it [GT results] came back that I didn’t carry the gene they’re looking for. –* Jody (73 years).

*So, it [EC] was a random cancer. So, it’s not genetically predisposed. No, it’s not related to the Lynch syndrome.* – Holly (57 years).

The remaining participants described their GT result as inconclusive or not definitive for LS.

*They saw the cells or whatever it is and kind of came back with this inconclusive [GT] result for me.* – Riley (43 years).

*So, they’ve [GC] given me the clinical diagnosis [of LS] but, it is not definitive and that’s where I suppose I’m in the interim of whether I have Lynch syndrome and if I do not.* – Kylie (58 years).

*As things stand at the moment, you haven’t got it [LS] but, we are not saying a 100% for sure that you definitely don’t have it because, there is information we don’t know and with research going on, that may change later.* - Frankie (53 years).

Many participants’ interpretations appeared to be guided by the HCP’s explanations.

*They [HCP] just told me that I didn’t have the Lynch syndrome.* – Tessa (67 years).

*They [HCP] said it [GT results] came back negative and that we don’t know the reasons why it has come back negative.* – Stella (74 years).

Some participants who reported an inconclusive/not definitive result for LS were confused regarding their GT results.

*I know there was at one point, I thought it was all fine and then at another point, I thought I was told that it [GT] was inconclusive and there was something that was not quite normal. There was some aberration or abnormality it is showing in the blood test. So, they [GC] couldn’t clearly say there was no Lynch syndrome.* – Amy (74 years).

#### Psychosocial impacts

Most participants who interpreted their GT result as negative, or negative for LS, reported feeling happy and relieved with the result. Some of them also mentioned that the results did not affect their daily routine.

*How I feel about the [GT] result? Very happy I suppose. I felt relieved.* – June (66 years).

*It [GT results] hasn’t had any effect [on daily life] whatsoever. As I said, I would have had if it was positive but, being negative, no. Absolute no.* – Teresa (64 years).

Some of the participants who understood their GT result as inconclusive/not definitive for LS reported experiencing negative responses such as anger, frustration, or disappointment from the result.

*To get in there and for it [GT result] to be inconclusive, it just felt cruel. (…*) *I felt quite cheated.* – Riley (43 years).

A few other participants who understood their GT result as inconclusive/not definitive for LS reported no negative psychosocial impacts of GT.

*I’m not concerned in the least [about GT results]. I’m very interested more so in genetics. (…) It hasn’t impacted my life negatively.* – Kylie (58 years).

#### Future cancer risk perception for self and family

Participants’ perceptions of their (Table 2) and their family’s (Table 3) future cancer risk was influenced by their understanding of their GT results. Most participants who understood their GT result as negative for LS believed that they were at population risk of future cancers, while a few others were not aware or could not recall their future cancer risks. They perceived their family to be at a population risk of future cancer or otherwise based future risks on their family history of cancer.

**Table 2:** Quotes representing participants’ perception of their future cancer risk

Interpretation of genetic test result	Cancer risk perception	Representative quote
Negative for LS	Population risk	*I believe that my risk [of cancer] is no more, or no less than anybody in the population.* - Teresa (64 years)
		*Because I didn’t have the Lynch syndrome, I would think that I’ve got just the same amount of risk as the other people [in the population].* – Tessa (67 years)
Inconclusive/ not definitive for LS	Higher risk than population	*Probably at a higher [than population] risk. It’s always a worry at the back of your head. – Amina (59 years)*
	Slightly higher risk than population	*I think it [future cancer risk] may be slightly higher.* – Reeta (51 years)
	Population level risk	*Oh, I don’t feel that it is any stronger. I mean the general population. I don’t think I’m at greater risk than anyone else [in the population].* – Amy (74 years)

*Abbreviations*: *LS* Lynch syndrome

**Table 3:** Quotes representing participants’ perception of their family members’ future cancer risk

Interpretation of genetic test result	Cancer risk perception for family	Representative quote
Negative for LS	Population risk	*I think we are all fairly baseline, my immediate family, and my extended family on both my mother’s and father’s side.* – Emilia (73 years)
	Risk due to family history of cancer	*As I said there was quite a bit of cancer in the family and so yeah, I think that contributes [to future cancer risk for family].* – Teresa (64 years)
Inconclusive/ not definitive for LS	Increased risk	*Both of the children could have some increased, potentially some increased risk. –* Riley (55 years)
	Slightly higher risk	*They said that my sister is at a slightly higher risk for what I’ve had* – Frankie (53 years)
	Risk of Lynch syndrome-type cancer	*They [family members] are at risk of having a Lynch syndrome type disease because of the genes involved in it.* – Kylie (58 years)

*Abbreviations*: *LS* Lynch syndrome

Most of the participants who stated they had received an inconclusive/not definitive for LS result from GT believed they were at a slightly higher or higher risk of future cancers than the general population, while a few reported they were at population risk. These participants also perceived their family to have a higher or slightly higher risk of future cancers, and a few reported their family to have a risk of LS-type cancer.

### Theme 2: Cancer risk-reducing behavior is independent of GT results

All EC patients were treated by surgical intervention and as a result they did not require ongoing screening of the uterus and/or ovaries. However, there were inconsistencies in CRC screening advice reported by participants. Participants discussed several motivations for cancer screening adherence.

#### Surgery to treat EC and prevent ovarian cancer risk

All but one participant reported undergoing a total hysterectomy and bilateral salpingo-oophorectomy and the other participant reported she had only her uterus removed.

#### Inconsistencies in CRC screening advice reported

There were inconsistencies in CRC screening advice that participants recalled being given, ranging from no advice, to having annual colonoscopies (Table 4). There were similar numbers of participants within each recommendation category. Only a few participants reported being advised to undertake annual or biennial colonoscopies, which is recommended for individuals with LS. Only one participant reported being advised to have gastroscopies for gastric cancer screening. One participant reported being advised to consume aspirin to reduce CRC risk.

**Table 4:** Quotes representing varied reported CRC screening recommendations

Reported CRC screening recommendation	Representative quote
No colonoscopy advice	*Interviewer: Did you receive any cancer screening advice based on your genetic test results from your genetic counsellor?* *No, I didn’t, actually.* – Riley (43 years)
One-off colonoscopy	*I had a colonoscopy last year. Well, I think they [gastroenterologist] told me that I didn’t really need to do another one at the moment.* – Tessa (67 years)
Colonoscopy once in 5 years	*… [advised] just to have a colonoscopy, which I did and, the surgeon told me that I didn’t need to have another one for five years.* – Stella (73 years)
Colonoscopy once in 3 years	*Well, the gastroenterologist recommended another one [colonoscopy] in three years’ time. So, I imagine that it would be a three-yearly thing to do.* – Amy (74 years)
Colonoscopy once in 1–2 years (LS screening recommendation)	*So, now I just do a colonoscopy every year, every twelve months*. – Amina (59 years)

*Abbreviations*: *LS* Lynch syndrome

#### Motivations to regularly pursue CRC screening

Of those participants who recalled receiving CRC screening recommendations, almost all adhered to these recommendations. Participants discussed several motivations for cancer screening adherence, including reducing the risk of cancer, early detection, wanting to live longer or not wanting to die young, HCP recommendation, and not affecting family members by remaining healthy (Table 5). There was an increased awareness of the benefits of cancer screening and adherence among participants with a healthcare background or experience of multiple cancers in the family.

*I just think working in that environment [as a nurse] makes you aware and you have to take your life into your own hands really. If somebody suggests something, just do it.* - June (67 years).

*My brother passed away, twenty years ago with a possible diagnosis of a primary in the bowel, (…) and so then, my siblings and I, my parents when they were alive, we have all had three-yearly colonoscopies and our families, as they’ve grown older, have also had colonoscopies.* – Emilia (73 years).

**Table 5:** Quotes representing several motivations to cancer screening and adherence

Motivations to cancer screening and adherence	Representative quotes
Prevention, early detection, or reduction of risk of any future cancers	*Oh, I think over the years it was to try and avoid any cancer for myself I suppose because of the family history and various family members that were having a cancer diagnosis. So, the motivation was to try and avoid having any very late diagnosis of cancer.* – Emilia (73 years)
	*I think I’ve got a better chance of finding it [cancer] earlier because I’m getting checked regularly.* – June (67 years)
	*Knowing like, being a nurse, I know that you can’t be complacent with these things and your genetic health is out of our hands. So, if I was to ignore the advice that I’ve been given and find that I’ve had a cancer that I could have intercepted early, I would be very disappointed in myself for being complacent.* – Kylie (58 years)
Wanting to live longer or not wanting to die young	*I want to get to old age. I don’t want to die young. (…) Yeah, I just want to, my motivation is to live not only a long life, but to have good quality of life.* – Tessa (67 years)
	*I want to live to I’m a hundred. I’ve always wanted. I got a life to live, lot of things I want to do.* – Amina (59 years)
Healthcare professional recommendation	*That’s just, I follow the doctor’s advice on that one. I rather have it, yearly screening tests. That’s essentially testing for cancer, so why wouldn’t we? Why wouldn’t I keep an eye on it? I think I’m very practical in that sense.* – Reeta (51 years)
	*Basically, anything the doctor says is a good idea, as long as it [cancer screening] makes sense to me, and it is not a crack pot idea, I’m happy to do it.* – Frankie (53 years)
Not affect family members	*I’d like to be healthy and to make sure nothing will affect my children or grandchildren.* – Rebecca (75 years)

#### Family cancer screening advice reported by participants

Only one-third of the cohort reported receiving CRC screening advice for their family members based on their GT results. Very few participants recalled the recommended frequency of CRC screening for their family.

*They told me that they have to have colonoscopies, especially my brothers, my sister and daughter. (…) He [son] has got the recommendation too.* – Rebecca (75 years).

*They suggested he [son] have a one-off colonoscopy.* – Stella (74 years).

Over one-third of the cohort did not recall receiving any cancer screening management advice for their family members. No participant recalled receiving advice on chemoprevention or risk-reducing surgery to manage family members’ future cancer risks.

### Theme 3: Family communication of GT results and cancer risk management

#### Family communication of GT results

Almost all participants communicated their understanding of their GT results to their immediate family member(s), including children, parents, or siblings, irrespective of their interpretation of the GT result as negative or inconclusive/not definitive for LS. Many participants also disclosed their GT result information to extended family members.

*Yes, they’re [immediate family] all aware of that [GT result] and the fact that my [genetic] test was clear. I kept them informed all the way through.* – Jody (73 years).

*Yes, with a couple of cousins, a few aunts and uncles because, my mom put all those details in a letter she sends out to everybody. All the family would have got the information [regarding GT results].* – Frankie (53 years).

#### Family communication of cancer risk management advice

Many participants who received cancer risk management advice for their family communicated this information. However, some participants who received cancer risk management advice did not pass this on.

*When I had the [genetic] testing, they [GC] suggested that he [son] should have a colonoscopy. I showed him the letter that they sent me.* – Stella (74 years).

#### Reasons for disclosure and non-disclosure of GT results and cancer risk management advice

Participants discussed several motivations for family communication, such as benefit to family, open relationships, and utility of communication due to multiple cancers in the family.

*Anything I find out I pass on and I hope that they’ll [family] be able to benefit from the information. I feel quite excited being able to help.* – Jess (72 years).

*The nieces, I have a pretty open relationship with them. They’re very easy to talk to, so I talked to them about it [cancer risk management advice].* – Amina (59 years).

Participants reported several reasons for non-disclosure of GT results and cancer risk management advice to family, including understanding the GT result to be negative or inconclusive for LS, cancer risk not being increased for family members, and estrangement.

*Well, my extended family live overseas. If I’d had Lynch disease, I think that [family communication] would’ve been different.* – June (66 years).

*I just didn’t think it [communication with extended family] was necessary because I was negative, and there really wasn’t enough high risk [of cancers].* – Teresa (64 years).

Another reason for non-disclosure included first-degree relatives being too young.

*I’ve not [spoken to the children], we have talked about it but, not anything specific. I’m sort of waiting for them to become adults. -* Riley (43 years).

## Discussion

This is the first study to specifically explore EC patient experiences of receiving an SLS diagnosis. We found that participants’ interpretation of their GT result varied and influenced the psychosocial implications of GT and their perceptions of their and their family’s future cancer risk. However, participant cancer screening behaviors were independent of their GT result interpretation, with almost all adhering to advice received. Almost all participants communicated their GT result to immediate family members. However, communication of family cancer risk management advice was more limited, mostly due to not receiving family screening advice.

### GT result interpretation

Findings from our study indicated that participants either considered themselves negative for LS or inconclusive/not definitive for LS. These findings are similar to Katz, Burton-Chase [19] where 41% of SLS-CRC patients understood their result to be negative or a VUS. However, the same study found that 37% of participants understood they were positive for LS, which differed in our study. The varied interpretations could be due to the different participant cancer types, differences in communication of results and the differences in healthcare settings and participant demographics. We had an all-female cohort and excluded women with a VUS finding from GT, whereas Katz, Burton-Chase [19] included males and females with CRC and 35% of participants had a VUS. Another possible explanation could be the inconsistent knowledge and understanding of SLS among HCPs, such as oncologists, gynecological oncologists, and surgeons [30].

### Psychosocial impacts of GT

Many participants who interpreted their result as negative for LS expressed happiness and relief. Those who interpreted their result as inconclusive/indeterminate for LS, were more likely to express negative psychosocial responses, such as frustration, disappointment or anger. These psychosocial responses may be linked to the uncertainty and cancer worry previously reported with an SLS diagnosis [19, 20, 31]. Solomon, Harrington [31] also reported variable psychosocial responses of SLS individuals with a VUS GT finding, such as relief, disappointment, and shock. Consistent with this, many of these participants also perceived themselves to be at higher risk of future cancers compared with participants who perceived their result as negative.

### Future cancer risk perception

Most participants in this study who understood their result as negative for LS thought they had a population or low(er) risk of future cancers as their perceived negative GT result had given them a false assurance that they did not have an inherited cancer etiology. This aligns with Grover, Stoffel [32] whose study found that 48% of SLS individuals underestimated their future cancer risk. Family history of multiple cancers sometimes informed cancer risk perception among CRC patients [33], which was also observed among our cohort of EC patients. This finding was also similar to Katz, Burton-Chase [19] who found that 68% of participants believed they had a higher risk for LS based on their personal and family history of cancers.

### Cancer risk-reducing behavior

There was an inconsistency in CRC screening advice reported by participants. Consequently, only a few participants pursued CRC screening at the frequency recommended for LS. This contrasts with studies involving a majority of SLS-CRC patients [19, 20], which reported 48–76% participants complied to annual-biennial colonoscopies. This discrepancy may be explained by the practice of regular colonoscopies required for CRC patients as part of their post-operative follow-up care [34], regardless of their GT result. Another reason for this discrepancy may be the lack of SLS cancer risk management guidelines in Australia, resulting in variations in clinical practices across jurisdictions/services. The US, on the other hand, have guidelines that recommend SLS individuals, and their first-degree relatives undertake LS cancer screening recommendations, while SLS individuals with biallelic somatic mutations are managed based on their personal and family history of CRC [17, 18].

The high compliance of EC-SLS patients with CRC screening recommendations was an unexpected finding in our study. This was surprising given many participants understood they had received a negative GT result for LS and perceived themselves to be at population risk of future cancers. Regular CRC screening in our cohort was motivated by HCP advice and a desire to reduce the risk of future cancers. Some individuals were further influenced by their family history of cancer or being employed as HCPs. This aligns with previous studies [35, 36] which showed that a family history of cancer motivated individuals to remain compliant with recommended cancer screening. Increased breast cancer screening adherence has also been reported among healthcare workers [37]. A previous study also found that women with uninformative GT results for *BRCA* genes believed in undergoing cancer screening despite a lower perceived risk of a deleterious variant [38], consistent with our findings.

Gastroscopy advice was not routinely provided to participants in our cohort. This contrasts with findings of Katz, Burton-Chase [19], who reported that many SLS-CRC participants (56%) undertook stomach cancer screening. This might be because, in Australia, biennial gastroscopies are only recommended to individuals with LS who have a family history or high ethnic risk of stomach cancer [8].

Literature cites the utility of aspirin in reducing the risk of CRC [39–41], and Australian guidelines recommend individuals with LS or a moderately increased risk of CRC to consume low-dose aspirin from age 30 and 50–70, respectively [8, 42]. However, only one participant recalled receiving advice to take aspirin as a CRC preventative measure, suggesting HCPs may not be applying LS guidelines to EC-SLS patients.

None of the family members of our participants were advised to pursue LS risk management recommendations, contrasting Katz, Advani [21] who reported that several family members of SLS-CRC patients followed LS screening recommendations [21]. Studies have found that family members of SLS patients are at an increased risk of LS-associated cancers [10, 16]. Hence, there is good reasoning for Australian risk management guidelines for SLS individuals and their families. In the US and UK, guidelines recommend that SLS-CRC individuals undergo tumor genomic sequencing to re-classify the 50–75% of cases with sporadic cancers resulting from biallelic somatic mutations in an MMR gene [17, 43–46]. Upon reclassification, tailored cancer risk management recommendations are available for individuals with sporadic cancer [4]. Thus, the development of guidelines for EC-SLS individuals is also warranted to better manage and support such individuals.

### Family communication

Almost all participants communicated their GT results to their immediate family, while many also discussed this with extended family members. However, communication of cancer screening recommendations was limited, mostly because they had not received family screening advice. The main reason participants communicated this information with family, was to reduce their risk of a cancer in the future. Some reasons for non-disclosure of information included interpretation of GT results as negative or inconclusive for LS, cancer risk not being perceived as increased significantly and estrangement. These findings augment the results of Katz, Burton-Chase [19] who reported estrangement, old age and assumption of communication through other relatives as reasons for non-disclosure of information to family. Moreover, only some participants reported receiving information regarding CRC screening for their family members based on their GT results. This lack of advice is a potential barrier to the screening of at-risk family members, given communication is heavily reliant on the patients themselves [47].

### Practice implications

Findings from our study highlight that participant compliance with cancer screening recommendations does not eliminate the importance for participants to accurately interpret their GT results, because this interpretation influences their cancer risk perception for themselves and their family, as well as their [motivation for] communication of this risk to family members. Hence, it is instrumental for GCs and HCPs to clearly convey tumor and germline results to SLS patients [19]. Follow-up calls or letters after 12 months of GT result disclosure can also be useful to clarify any patient misconceptions or concerns [48].

We found inconsistencies in CRC risk management advice reported by participants for themselves and family members. This could be overcome by uniform national and international cancer screening guidelines for EC-SLS individuals, including recommending genomic sequencing of EC tumors to identify patients with double somatic MMR mutations, a non-inherited cause of tumor MMR-deficiency.

### Study limitations and research recommendations

This qualitative study is the first to explore EC patients’ experiences of a SLS diagnosis. Purposive sampling and multi-site recruitment ensured diversity in participant demographics and experiences. However, women of Caucasian background and who worked as HCPs were over-represented. Thus, findings should not be generalized to all populations. Additional studies that explore the experiences of EC-SLS patients and their family members in other jurisdictions would be helpful to support and augment these findings. Our study was designed to capture a wide range of experiences, including genetic counselling at different cancer genetics services with different GCs. Capturing this variation and range of experiences related to current practice was important for the future creation and implementation of standard processes and Australian guidelines. Isolating the heterogeneity observed in psychosocial and behavioral responses caused by genetic counselling differences from the individual patient’s interpretations and responses and the compounding effect of this is difficult with this study design. Future studies which test the impact of standardized counselling informed by the findings of this study, on women’s understanding of their test results and subsequent health behavior may be useful to develop guidance for how GCs and other HCPs can optimally manage and support such individuals.

## Conclusion

This study reinforces the importance of clear and accurate communication of tumor and germline GT results by HCPs despite high participant compliance with cancer screening recommendations. This is because participant understanding influences their psychosocial responses and cancer risk perception. It may also affect the disclosure of GT results and cancer screening recommendations to family members. The lack of family communication, together with the reported lack of family screening advice provided by HCPs could result in sub-optimal protection of these at-risk individuals. Findings highlight variability in the psychosocial and behavioral responses associated with EC-SLS, which is good reasoning for the development of Australian risk management guidelines to improve consistency in SLS individuals and their families. Study findings also provide an understanding of how HCPs can better manage and support such individuals.

## Supplementary Information

**Additional file 1.**

## Data Availability

The data that support the findings of this study are available on request from the corresponding author. The data are not publicly available due to privacy or ethical restrictions.

## Abbreviations

LS: Lynch Syndrome
MMR: Mis-match repair
CRC: Colorectal cancer
EC: Endometrial Cancer
GT: Genetic testing
IHC: Immunohistochemistry
SLS: Suspected Lynch Syndrome
VUS: Variant of Uncertain significance
HCPs: Healthcare Professional(s)
GCs: Genetic Counsellor(s)
ANGELS: Applying novel genomic approaches to early-onset and suspected Lynch syndrome colorectal and endometrial cancers
LOE: Loss of Expression

## References

[1] Lynch HT, Smyrk T (1996). Hereditary nonpolyposis colorectal cancer (Lynch syndrome). An updated review. Cancer.

[2] Cohen SA, Leininger A (2014). The genetic basis of Lynch syndrome and its implications for clinical practice and risk management. Appl Clin Genet.

[3] Lynch HT, Shaw MW, Magnuson CW, Larsen AL, Krush AJ (1966). Hereditary factors in cancer. Study of two large midwestern kindreds. Arch Intern Med.

[4] Vasen HFA, Blanco I, Aktan-Collan K, Gopie JP, Alonso A, Aretz S (2013). Revised guidelines for the clinical management of Lynch syndrome (HNPCC): recommendations by a group of European experts. Gut.

[5] Giardiello FM, Allen JI, Axilbund JE, Boland CR, Burke CA, Burt RW (2014). Guidelines on genetic evaluation and management of Lynch syndrome: a consensus statement by the US multi-society task force on colorectal cancer. Gastroenterology.

[6] Latham A, Srinivasan P, Kemel Y, Shia J, Bandlamudi C, Mandelker D (2019). Microsatellite instability is associated with the presence of Lynch syndrome pan-cancer. J Clin Oncol.

[7] Samadder NJ, Smith KR, Wong J, Thomas A, Hanson H, Boucher K (2017). Cancer risk in families fulfilling the Amsterdam criteria for Lynch syndrome. JAMA Oncol.

[8] EviQ: MMR genes (Lynch syndrome) – risk management Australia: Cancer Institute NSW; 2009 [updated 06/17/2019. 1410 v.9:[Available from: https://www.eviq.org.au/cancer-genetics/adult/risk-management/1410-mmr-genes-lynch-syndrome-risk-management

[9] Carethers JM (2014). Differentiating Lynch-like from Lynch syndrome. Gastroenterology.

[10] Rodríguez-Soler M, Pérez-Carbonell L, Guarinos C, Zapater P, Castillejo A, Barberá VM (2013). Risk of cancer in cases of suspected Lynch syndrome without germline mutation. Gastroenterology..

[11] Hampel H, Pearlman R, de la Chapelle A, Pritchard CC, Zhao W, Jones D (2021). Double somatic mismatch repair gene pathogenic variants as common as Lynch syndrome among endometrial cancer patients. Gynecol Oncol.

[12] Wang T, Lee LH, Vyas M, Zhang L, Ganesh K, Firat C (2019). Colorectal carcinoma with double somatic mismatch repair gene inactivation: clinical and pathological characteristics and response to immune checkpoint blockade. Mod Pathol.

[13] Hemminger JA, Pearlman R, Haraldsdottir S, Knight D, Jonasson JG, Pritchard CC (2018). Histology of colorectal adenocarcinoma with double somatic mismatch-repair mutations is indistinguishable from those caused by Lynch syndrome. Hum Pathol.

[14] Bucksch K, Zachariae S, Aretz S, Büttner R, Holinski-Feder E, Holzapfel S (2020). Cancer risks in Lynch syndrome, Lynch-like syndrome, and familial colorectal cancer type X: a prospective cohort study. BMC Cancer.

[15] Gordhandas S, Kahn R, Maddy BP, Nelson BB, Askin G, Christos PJ (2019). Lynch-like syndrome in endometrial cancer: features of a growing population. J Clin Oncol.

[16] Picó MD, Castillejo A, Murcia Ó, Giner-Calabuig M, Alustiza M, Sánchez A (2020). Clinical and pathological characterization of Lynch-like syndrome. Clin Gastroenterol Hepatol.

[17] Gupta S, Weiss JM, Axell L, Burke CA, Chen L, Chung DC (2021). National Comprehensive Cancer Network (NCCN) clinical practice guidelines in oncology: genetic/familial high-risk assessment: colorectal National Comprehensive Cancer Network.

[18] Leggett B, Poplawski N, Rosty C, Norton I, Wright C, Aung KW (2017). Cancer Council Australia colorectal cancer guidelines working party. Guidelines: Colorectal cancer/Lynch syndrome. In: Clinical practice guidelines for the prevention, early detection and management of colorectal cancer.: Sydney: Cancer Council Australia.

[19] Katz LH, Burton-Chase AM, Advani S, Fellman B, Polivka KM, Yuan Y (2016). Screening adherence and cancer risk perceptions in colorectal cancer survivors with Lynch-like syndrome. Clin Genet.

[20] Omark J, Vilar E, You YN, Dunnington L, Noblin S, Stevens B (2020). Patients with unexplained mismatch repair deficiency are interested in updated genetic testing. Hered Cancer Clin Pract.

[21] Katz LH, Advani S, Burton-Chase AM, Fellman B, Polivka KM, Yuan Y (2017). Cancer screening behaviors and risk perceptions among family members of colorectal cancer patients with unexplained mismatch repair deficiency. Familial Cancer.

[22] Cachia M, Millward L (2011). The telephone medium and semi-structured interviews: a complementary fit. Q Res Org Manag.

[23] NVivo software Version 12: QSR International Pty Ltd. 2018 [Available from: https://www.qsrinternational.com/nvivo/trial.

[24] Nowell LS, Norris JM, White DE, Moules NJ (2017). Thematic analysis: striving to meet the trustworthiness criteria. Int J Qual Methods.

[25] Clarke V, Braun V (2018). Using thematic analysis in counselling and psychotherapy research: a critical reflection. Couns Psychother Res.

[26] Braun V, Clarke V (2012). Thematic analysis. APA handbook Res Methods Psychol.

[27] Braun V, Clarke V, Rance N. How to use thematic analysis with interview data (process research). The Counselling & Psychotherapy Research Handbook. 1 ed: Sage; 2014.

[28] Saunders B, Sim J, Kingstone T, Baker S, Waterfield J, Bartlam B (2018). Saturation in qualitative research: exploring its conceptualization and operationalization. Qual Quant.

[29] Soslow RA, Tornos C, Park KJ, Malpica A, Matias-Guiu X, Oliva E (2019). Endometrial carcinoma diagnosis: use of FIGO grading and genomic subcategories in clinical practice: recommendations of the International Society of Gynecological Pathologists. Int J Gynecol Pathol.

[30] Tan YY, Fitzgerald LJ (2014). Barriers and motivators for referral of patients with suspected lynch syndrome to cancer genetic services: a qualitative study. J Pers Med.

[31] Solomon I, Harrington E, Hooker G, Erby L, Axilbund J, Hampel H (2017). Lynch syndrome limbo: patient understanding of variants of uncertain significance. J Genet Couns.

[32] Grover S, Stoffel EM, Mercado RC, Ford BM, Kohlman WK, Shannon KM (2009). Colorectal cancer risk perception on the basis of genetic test results in individuals at risk for Lynch syndrome. J Clin Oncol.

[33] Robb KA, Miles A, Wardle J (2007). Perceived risk of colorectal cancer: sources of risk judgments. Cancer Epidemiol Biomarkers Prev.

[34] Colorectal Cancer: Follow-up care: ASCO Cancer. Net 2021 [Available from: https://www.cancer.net/cancer-types/colorectal-cancer/follow-care.

[35] Koenig J, Trees A (2006). Finding meaning in difficult family experiences: sense-making and interaction processes during joint family storytelling. J Fam Commun.

[36] Taylor DP, Cannon-Albright LA, Sweeney C, Williams MS, Haug PJ, Mitchell JA (2011). Comparison of compliance for colorectal cancer screening and surveillance by colonoscopy based on risk. Genet Med.

[37] Seah M, Tan S (2007). Am I breast cancer smart? Assessing breast cancer knowledge among healthcare professionals. Singap Med J.

[38] van Dijk S, Otten W, Timmermans DRM, van Asperen CJ, Meijers-Heijboer H, Tibben A (2005). What’s the message? Interpretation of an uninformative BRCA1/2 test result for women at risk of familial breast cancer. Genet Med.

[39] Rothwell PM, Wilson M, Elwin CE, Norrving B, Algra A, Warlow CP (2010). Long-term effect of aspirin on colorectal cancer incidence and mortality: 20-year follow-up of five randomised trials. Lancet.

[40] Burn J, Sheth H, Elliott F, Reed L, Macrae F, Mecklin J-P (2020). Cancer prevention with aspirin in hereditary colorectal cancer (Lynch syndrome), 10-year follow-up and registry-based 20-year data in the CAPP2 study: a double-blind, randomised, placebo-controlled trial. Lancet.

[41] Jenkins MA, Ait Ouakrim D, Boussioutas A, Hopper JL, Ee HC, Emery JD (2018). Revised Australian national guidelines for colorectal cancer screening: family history. Med J Aust.

[42] EviQ: Colorectal cancer (moderately increased risk) – risk management: Cancer Institute NSW; 2014 [updated 03/29/2019. 1425 v.6:[Available from: https://www.eviq.org.au/cancer-genetics/adult/risk-management/1425-colorectal-cancer-moderately-increased-risk#cancer-tumour-risk-management-guidelines.

[43] Monahan KJ, Bradshaw N, Dolwani S, Desouza B, Dunlop MG, East JE (2020). Guidelines for the management of hereditary colorectal cancer from the British Society of Gastroenterology (BSG)/Association of Coloproctology of Great Britain and Ireland (ACPGBI)/United Kingdom Cancer genetics group (UKCGG). Gut.

[44] Byers HM, Jacobson A, McFaddin AS, Ussakli CH, Newlin A, Stanich PP (2018). Postmortem somatic sequencing of tumors from patients with suspected Lynch syndrome has clinical utility for surviving relatives. JCO Precis Oncol.

[45] Salvador MU, Truelson MRF, Mason C, Souders B, LaDuca H, Dougall B (2019). Comprehensive paired tumor/germline testing for Lynch syndrome: bringing resolution to the diagnostic process. J Clin Oncol.

[46] O’Shea R, Rankin NM, Kentwell M, Gleeson M, Tucker KM, Hampel H (2021). Stakeholders’ views of integrating universal tumour screening and genetic testing for colorectal and endometrial cancer into routine oncology. Eur J Hum Genet.

[47] Leenen CH, Heijer M, van der Meer C, Kuipers EJ, van Leerdam ME, Wagner A (2016). Genetic testing for Lynch syndrome: family communication and motivation. Familial Cancer.

[48] Palmquist AEL, Koehly LM, Peterson SK, Shegog M, Vernon SW, Gritz ER (2010). “The Cancer bond”: exploring the formation of cancer risk perception in families with Lynch syndrome. J Genet Couns.

