# Supplementary material for: Heterogeneity in the psychosocial and behavioral responses associated with a diagnosis of suspected Lynch syndrome in women with endometrial cancer
Source: Hered Cancer Clin Pract. 2022 Jul 15;20:27. doi: 10.1186/s13053-022-00233-1 (PMC9284782; doi:10.1186/s13053-022-00233-1)
Supplement: Supplementary file 1 — Additional file 1. [file 13053_2022_233_MOESM1_ESM.docx]

**Conversational interview guide**

Patient ID -

*Introduction -*

Hello “________.” I am Sowmya Jonnagadla, a student researcher at the Victorian Comprehensive Cancer Centre. I would like to start off by thanking you for taking part in this study.

I am looking forward to hearing your experiences and feelings. This means, there are no right or wrong answers and what you truly feel, and experience is most important. This is because, the study intends to inform genetic counselling practices to provide better care for individuals, like yourself.

This interview will last for about 45 minutes to 1 hour. If a question is too sensitive, you can choose to skip it, or you can stop or take a break from the interview at any time. The interview will be audio-taped but, any published information will not have your name on it. If you want to talk more to someone independent about anything that we’ve brought up today, we can make referrals for you accordingly. Are you good to begin? Or would you like a second to catch a sip of water?

Wonderful, let’s get into it! Tell me about yourself.

*Understanding of cancer diagnosis-*

Can you please tell me what you understand about your cancer diagnosis?

Do you know anything about your specific type of uterine cancer?

What did genetic testing for you reveal?

What exactly do you mean by *“mutated gene/SLS/ inconclusive?”* *(mirror their language)*

Who or from where did you get all this information?

Can you tell what you understand about your family member’s risk of cancer, based on your genetic result?

*Perception of cancer –*

Did anyone talk to you about any other types of cancer and your risk of them?

How does your future cancer risk differ from that of other people?

*Psychosocial response to SLS diagnosis –*

How do you feel about your future cancer risk?

How do you feel about your genetic test result?

Describe your experience with genetic testing. This can include any good and bad experiences. *(In terms of daily life, perception, family dynamics, relationships, work/study, travel)*

How do these experiences make you feel?

Have you used any external psychosocial support since your diagnosis?

**(If yes)* What supports have been useful for you during this time? *(like psychologists, GP, community support, online/phone counselling, support groups, family, friends)*

Would you like to catch a breath for a second? Or can we continue?

*Cancer risk management and screening behaviours –*

Did you receive any cancer screening advice based on your genetic test result?

**(If yes)* What was the advice? *(different cancer screening)*

Who gave you that information? *(different health professionals/ internet)*

Did you receive any other advice regarding cancer prevention strategies? *(risk-reducing surgery/ lifestyle)*

Have you explored any other sources to obtain information or guidance on cancer prevention?

What cancer screening have you undertaken? *(different cancers)*

How often do you follow it?

Do you have less or more screening than the provided recommendations?

Why so?

Have you taken any other steps to reduce your cancer risk? *(lifestyle choices)*

What motivates you to take up cancer screening and these other changes to reduce cancer risk?

What do you feel are the barriers that stop you from taking action?

*Family communication of SLS diagnosis and cancer risk management advice –*

Have you communicated your genetic test results to your family?

**(If yes)* With whom? *(immediate/extended family)*

How did you convey your diagnosis/ genetic test result? *(letter from GCs)*

What were you told regarding your family members having genetic testing/ cancer screening?

**(If anything was told)* Have you communicated this information to them?

Did you face any challenges while informing your family members?

**(If yes)* What were the challenges?

What are the reasons for not communicating your diagnosis/ genetic test results with your family members?

*Conclusion –*

Is there anything else you would like to discuss about your cancer diagnosis or your genetic test result, that we missed?

In case you have any concerns or may require extra support, please contact Sharelle Joseland on ________.

Would you like to receive a summary of outcomes from this study?

Thank you once again for taking out your valuable time for this interview and for your patience. I highly appreciate your contribution to research in this field of cancer and genetics.

Hope you have a good day and great week ahead. Take care.

Bye.
